# Supplementary material for: Causal Relationship between Aging and Anorexia Nervosa: A White-Matter-Microstructure-Mediated Mendelian Randomization Analysis
Source: Biomedicines. 2024 Aug 16;12(8):1874. doi: 10.3390/biomedicines12081874 (PMC11351342; doi:10.3390/biomedicines12081874)
Supplement: Supplementary file 1 [file biomedicines-12-01874-s001.zip › Supplementary Figure/Figure S4/Singe SNP plot of MR analysis for TL to ubm-b-1650.pdf]

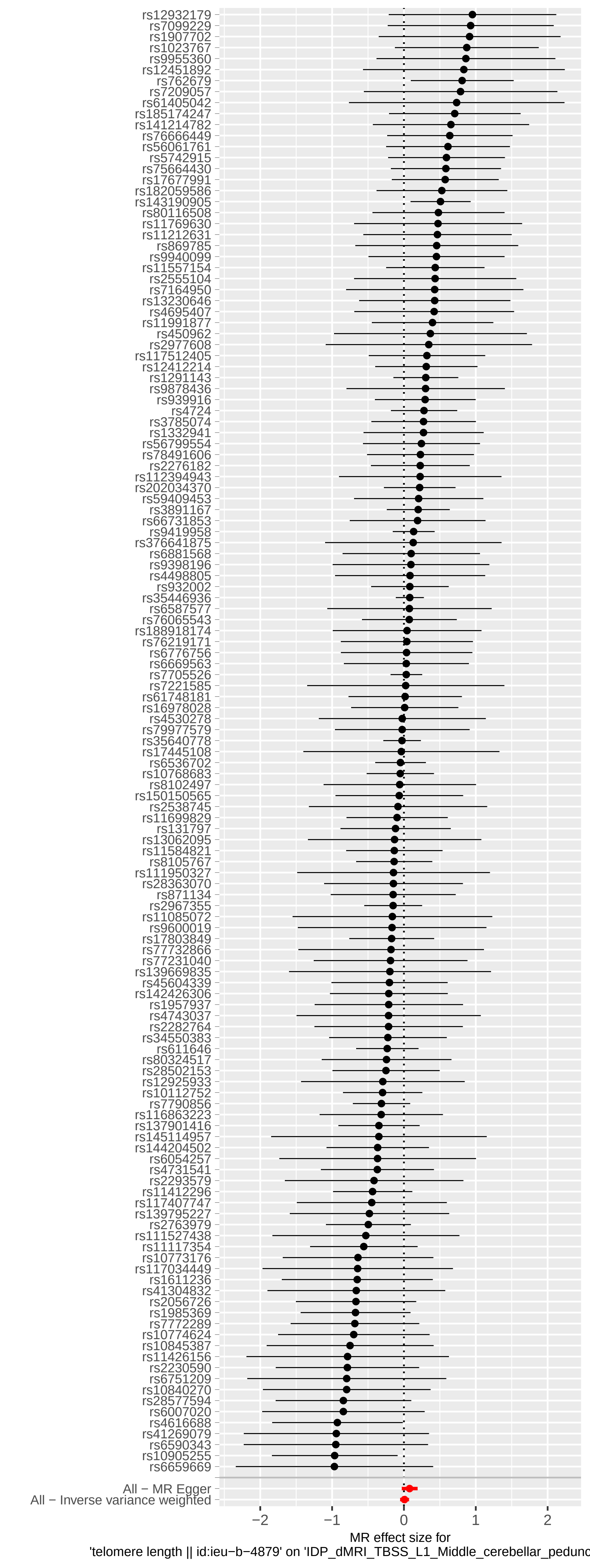
rs12932179
rs7099229
rs1907702
rs1023767
rs9955360
rs12451892
rs762679
rs7209057
rs61405042
rs185174247
rs141214782
rs76666449
rs56061761
rs5742915
rs75664430
rs17677991
rs182059586
rs143190905
rs80116508
rs11769630
rs11212631
rs869785
rs9940099
rs11557154
rs2555104
rs7164950
rs13230646
rs4695407
rs11991877
rs450962
rs2977608
rs117512405
rs12412214
rs1291143
rs9878436
rs939916
rs4724
rs3785074
rs1332941
rs56799554
rs78491606
rs2276182
rs112394943
rs202034370
rs59409453
rs3891167
rs66731853
rs9419958
rs376641875
rs6881568
rs9398196
rs4498805
rs932002
rs35446936
rs6587577
rs76065543
rs188918174
rs76219171
rs6776756
rs6669563
rs7705526
rs7221585
rs61748181
rs16978028
rs4530278
rs79977579
rs35640778
rs17445108
rs6536702
rs10768683
rs8102497
rs150150565
rs2538745
rs11699829
rs131797
rs13062095
rs11584821
rs8105767
rs111950327
rs28363070
rs871134
rs2967355
rs11085072
rs9600019
rs17803849
rs77732866
rs77231040
rs139669835
rs45604339
rs142426306
rs1957937
rs4743037
rs2282764
rs34550383
rs611646
rs80324517
rs28502153
rs12925933
rs10112752
rs7790856
rs116863223
rs137901416
rs145114957
rs144204502
rs6054257
rs4731541
rs2293579
rs11412296
rs117407747
rs139795227
rs2763979
rs111527438
rs11117354
rs10773176
rs117034449
rs1611236
rs41304832
rs2056726
rs1985369
rs7772289
rs10774624
rs10845387
rs11426156
rs2230590
rs6751209
rs10840270
rs28577594
rs6007020
rs4616688
rs41269079
rs6590343
rs10905255
rs6659669
All − MR Egger
All − Inverse variance weighted
−2
−1
0
1
2
MR effect size for
'telomere length || id:ieu−b−4879' on 'IDP_dMRI_TBSS_L1_Middle_cerebellar_pedunc
